# Supplementary material for: Metabolic and imaging phenotypes associated with RB1 and TP53 loss in prostate cancer
Source: Neoplasia. 2025 Oct 7;70:101235. doi: 10.1016/j.neo.2025.101235 (PMC12538569; doi:10.1016/j.neo.2025.101235)
Supplement: Supplementary file 1 [file mmc1.docx]

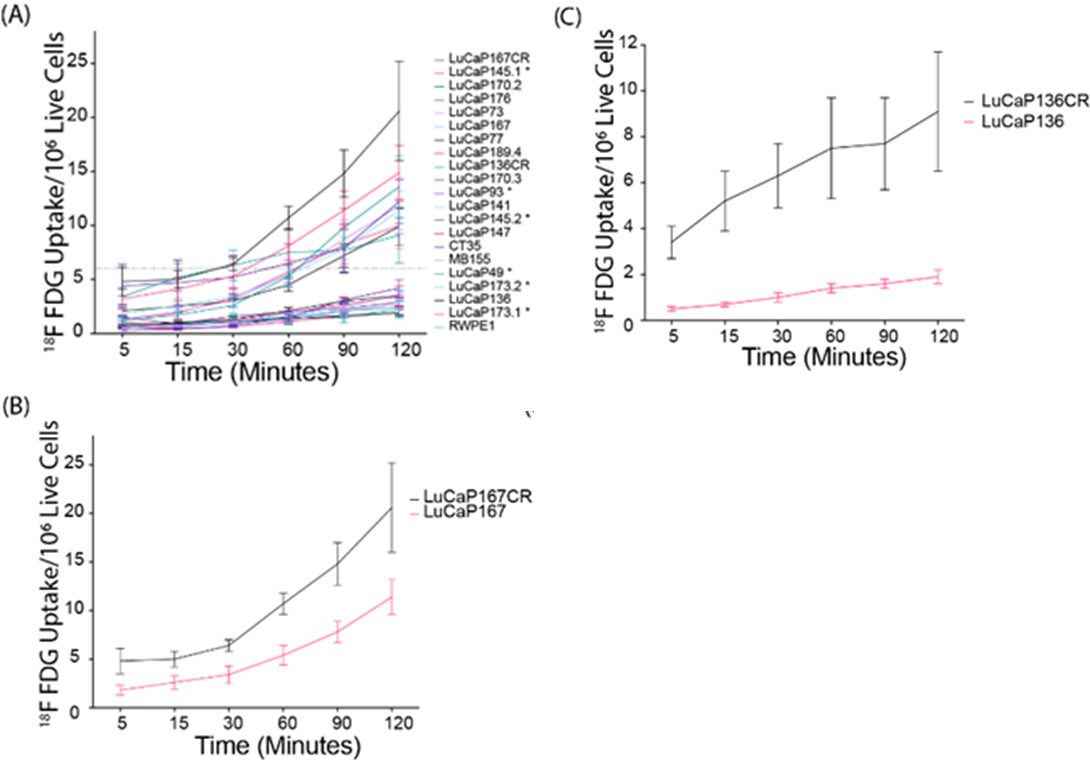


**Supplementary Figure 1.** **Adenocarcinoma, castration-resistant prostate cancer models display higher FDG uptake**. **(A)** FDG uptake over time per model; % uptake / 10^6^ live cells = [(cpm uptake) / (cpm total added) x (live cells total added)] x (10^6^live cells) x 100) for each timepoint. Line denoting the 6% FDG uptake indicates the separation between FDG-high models and FDG-low models. **(B, C)** FDG uptake over time in matched parental (castration-sensitive) and castration-resistant (CR) PDX-derived organoid models , where CR was selected via *in vivo* androgen deprivation (surgical castration of host mice).

**
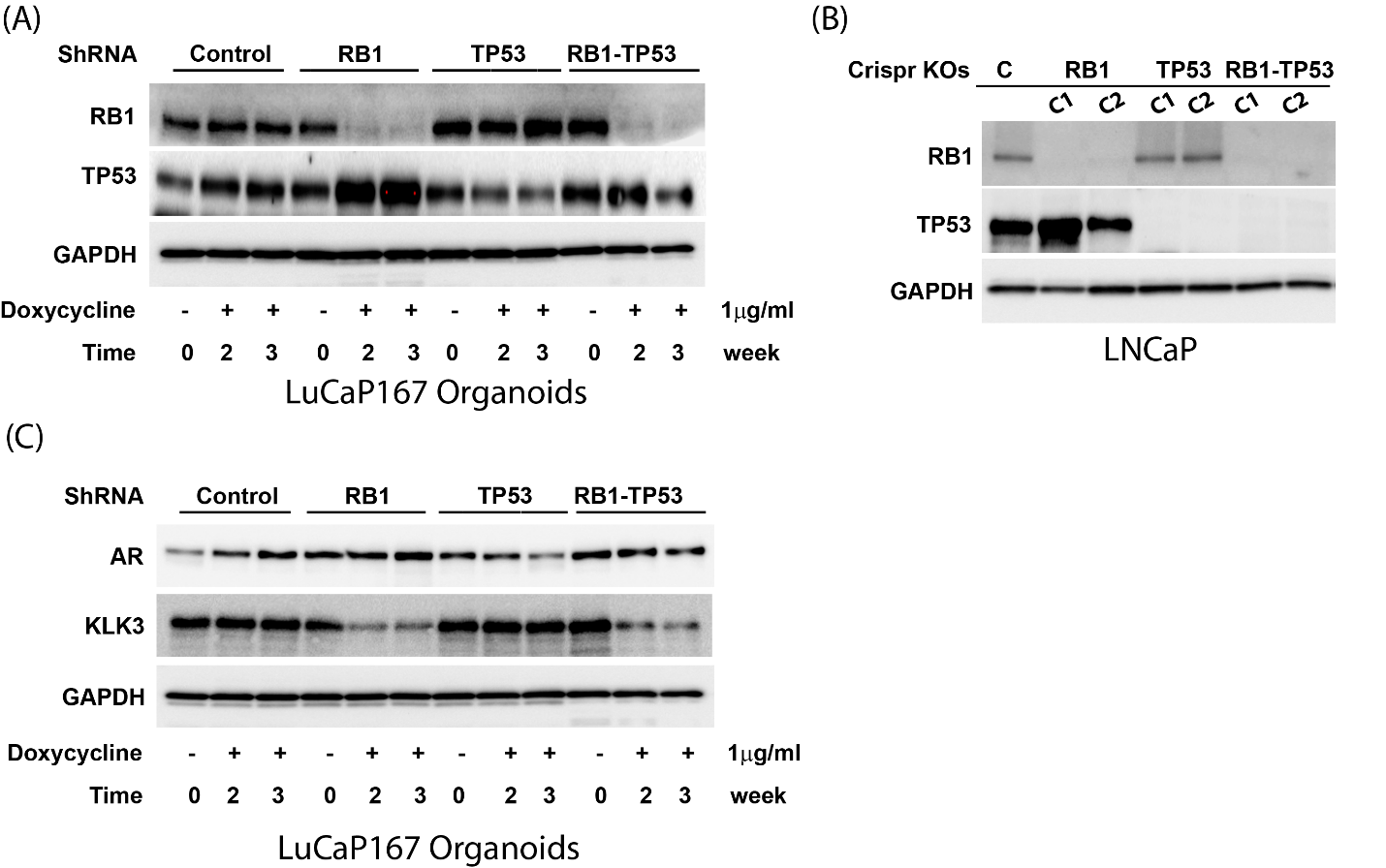
**

**Supplementary Figure 2.** ***RB1* and/ or *TP53* depletion maintains AR expression.** (**A)** Western blot showing RB1, TP53 and GAPDH protein expression in *RB1* and/or *TP53* altered LuCaP167 organoid cultures after 0, 2, or 3 weeks of ShRNA induction by doxycycline. **(B)** Western blot showing RB1, TP53 and GAPDH protein expression in *RB1* and/or *TP53* LNCaP Crispr KOs. **(C)** Western blot showing AR, KLK3 and GAPDH protein expression in *RB1* and/or *TP53* altered 167 LuCaP organoid cultures post 0, 2, or 3 weeks of doxycycline induction.

**
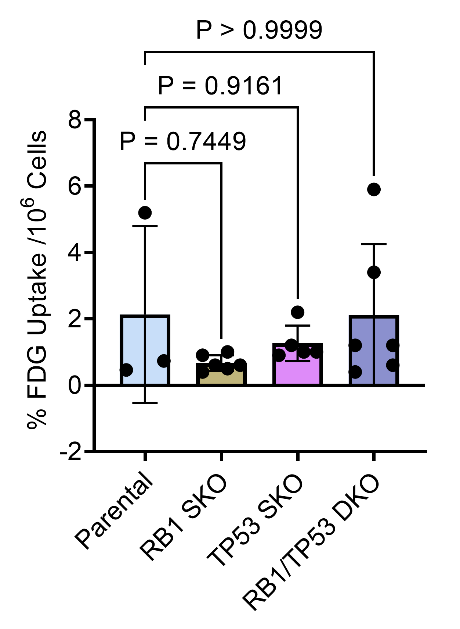
**

**Supplementary Figure 3.** ***RB1* and/ or*TP53* knockdown does not affect**: **^18^FDG^+^ uptake in LnCaP monoclonal Crispr knockouts.** ^18^FDG uptake per million cells after 120 minutes CRISPR/CAS9 knockout of *RB1* and/or *TP53*. Data is mean ± SD. One-way ANOVA test with Dunnett’s multiple comparisons test was performed using Prism 9.0. p= * (0.05), ** (0.01), *** (0.001).


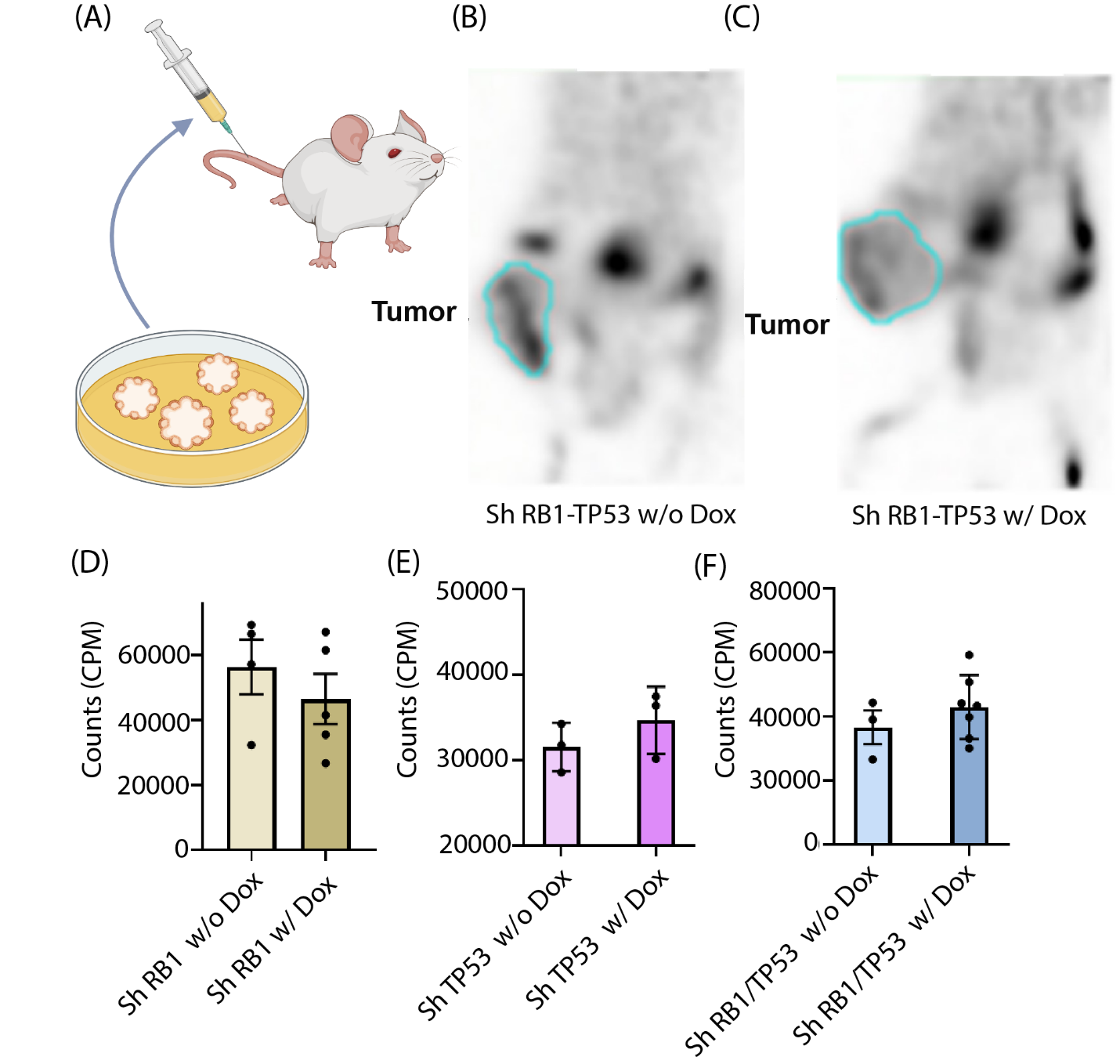


**Supplementary Figure 4.** ***RB1* and/ or*TP53* loss does not affect ^18^FDG^+^ uptake in a LuCaP 167 PDX model.** **(A)** Experimental setup. LuCaP 167 organoids were cultured in vitro, mixed with Matrigel, and implanted subcutaneously into NOD *scid* gamma (NSG) mice to create tumor xenografts. **(B and C)** ^18^FDG^+^-PET scan image showing total ^18^FDG^+^ activity present inside LuCaP 167 (PDX) mouse castration sensitive prostate cancer xenograft models with and without induction of shRNA targeting *RB1* and *TP53* by doxycycline. **(D, E, F)** Total amount of ^18^FDG^+^ calculated in scintillation counter from excised tumors with shRNA targeting *RB1* (n=5), *TP53* (n=3), *RB1/TP53* (n=7) induced by doxycycline, and their respective controls without doxycycline induction (n=4, 3, 3). Difference between control and knockdowns were not statistically significant. Data mean ± SEM, ns = not significant. Student t test was performed using Prism 9.0. p= * (0.05), ** (0.01), *** (0.001).


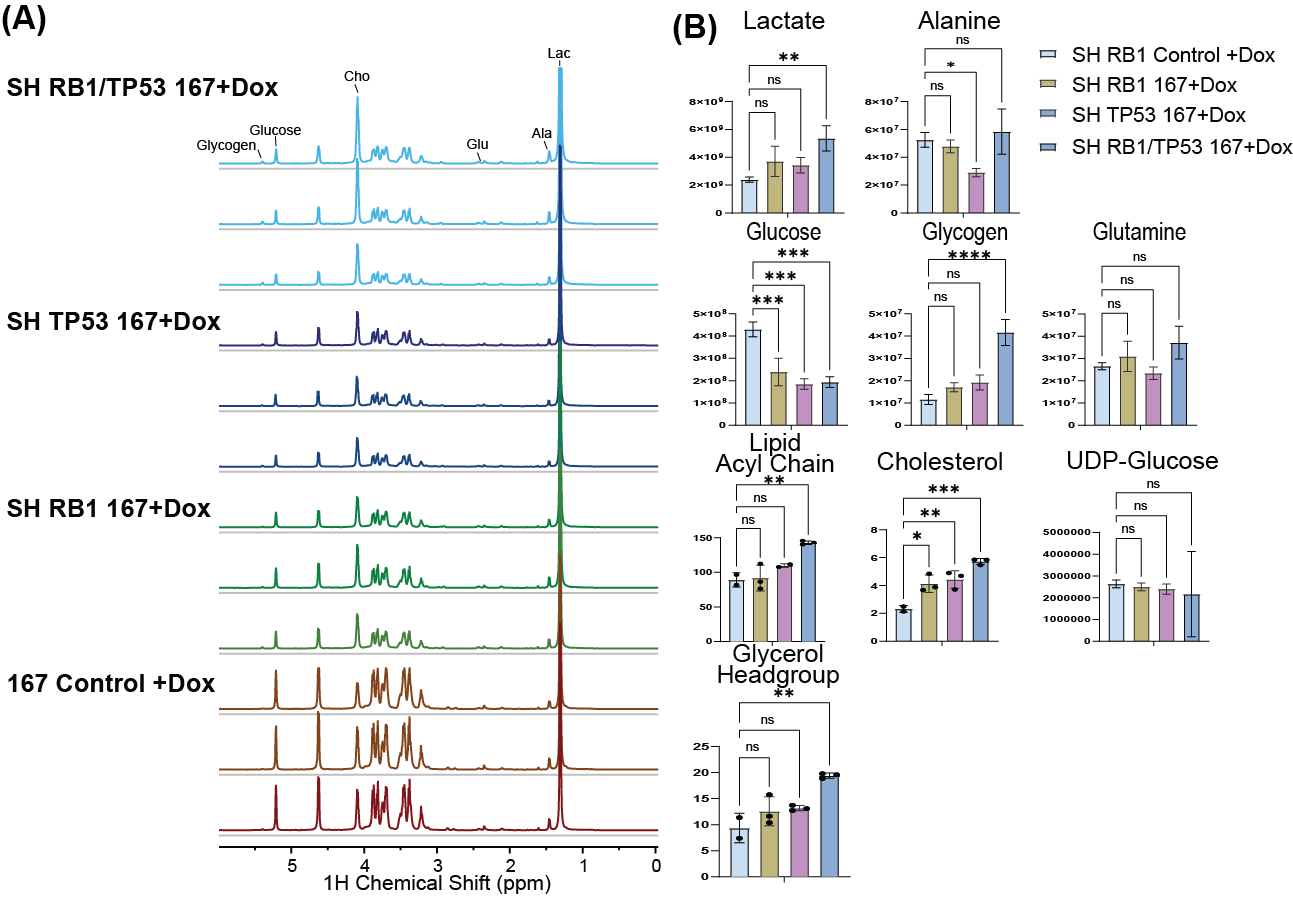


**Supplementary Figure 5. (A)** ^1^H-^13^C HSQC NMR spectra of the polar fraction of 2 million LuCaP 167 organoid cells. Peaks used for assignment of specific metabolites are labeled by arrows **(B)** Quantification of metabolites from the polar and non-polar fractions. Multiplicity corrected p-values are calculated form two-way ANOVA test with Tukey’s correction for multiple comparisons using Prism 9.0. p= * (0.05), ** (0.01), *** (0.001). Error bars indicate SD.


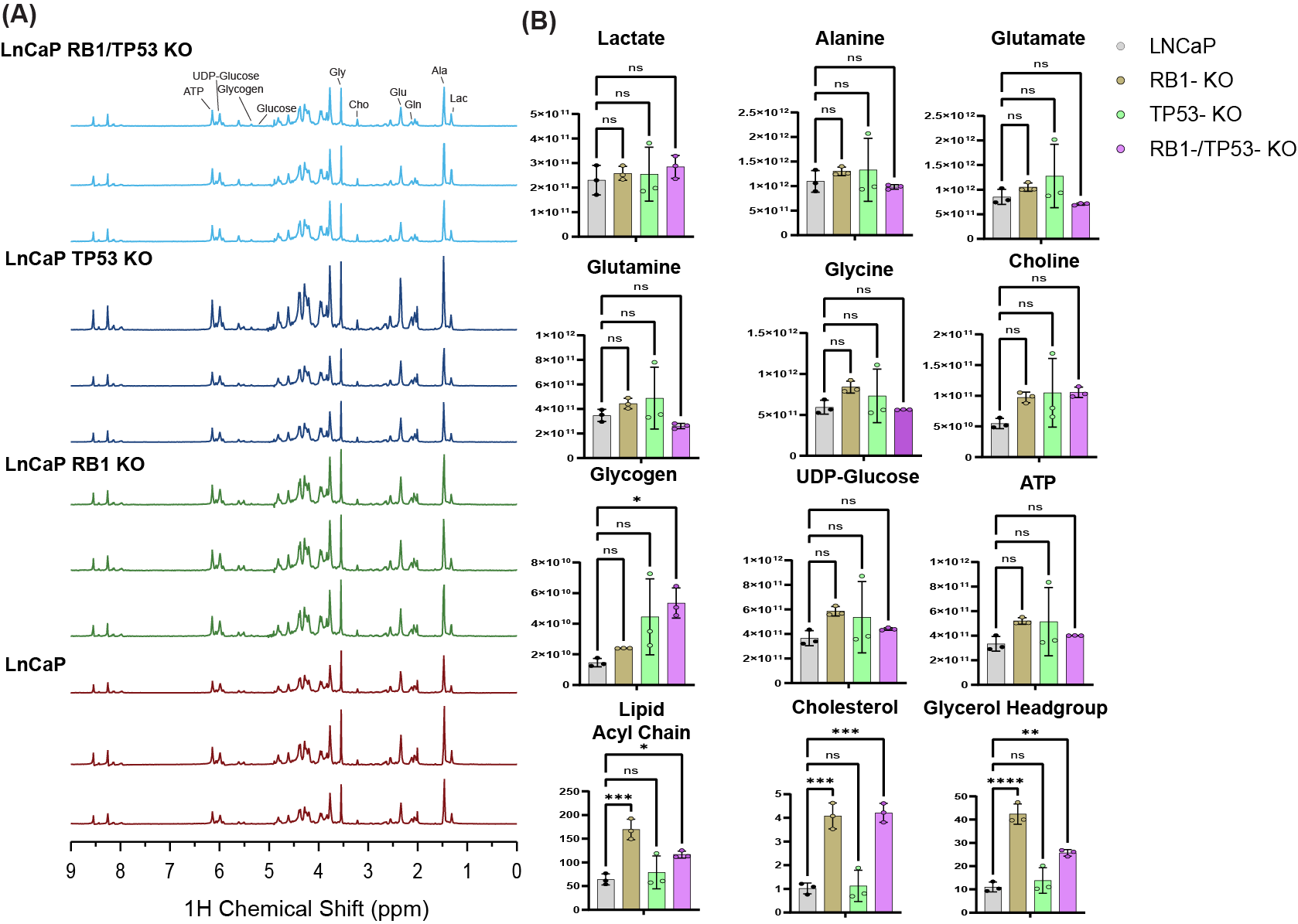


**Supplementary Figure 6. (A)** ^1^H-^13^C HSQC NMR spectra of the polar fraction of LNCaP monoclonal Crispr KOs cells normalized to protein concentration by BCA. Peaks used for assignment of specific metabolites are labeled by arrows **(B)** Quantification of metabolites from the polar and non-polar fractions. Multiplicity corrected p-values are calculated form two-way ANOVA test with Dunnet’s correction for multiple comparisons using Prism 9.0. p= * (0.05), ** (0.01), *** (0.001). Error bars indicate SD.


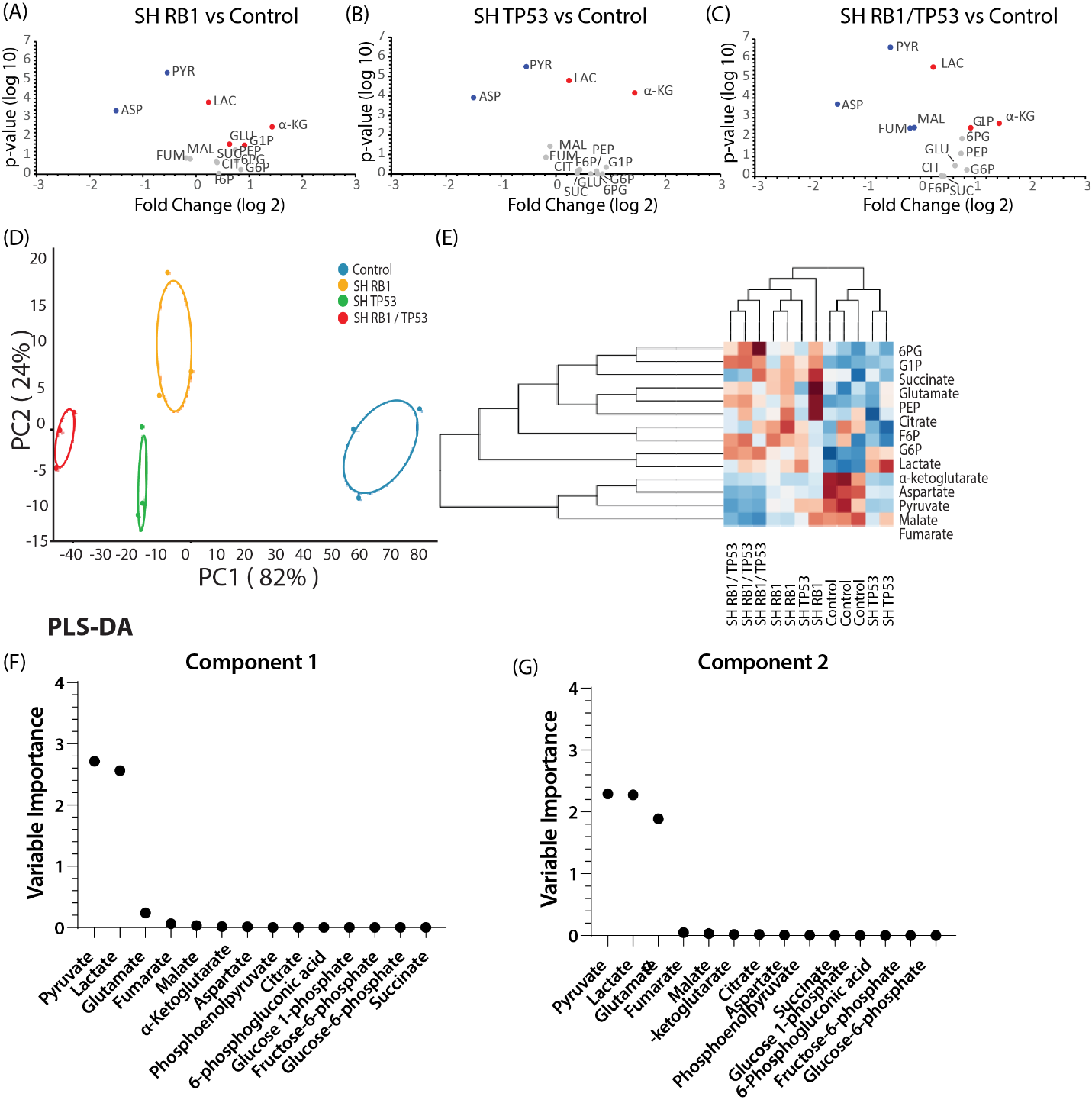


**Supplementary Figure 7****. Metabolite concentrations from IC-MS of LuCaP 167 organoids with shRNA knockdown of *RB1* and/ or *TP53* relative to controls without shRNA.** **(A to C)** Volcano plot of the log 2-fold change versus the associated p-value. Blue is significantly upregulated; red is significantly upregulated with **(D)** Principal component plot of the concentrations normalized to the total metabolite sum. The primary separation is the control samples from the RB1/TP53 depleted. **(E)** Heat map with hierarchal clustering **(F** and **G)** Variable importance for the first two components in a PLS-DA classification model for the four samples. The normalized concentrations of lactate, pyruvate, and fumarate are sufficient to separate the four samples (R^2^=0.572).


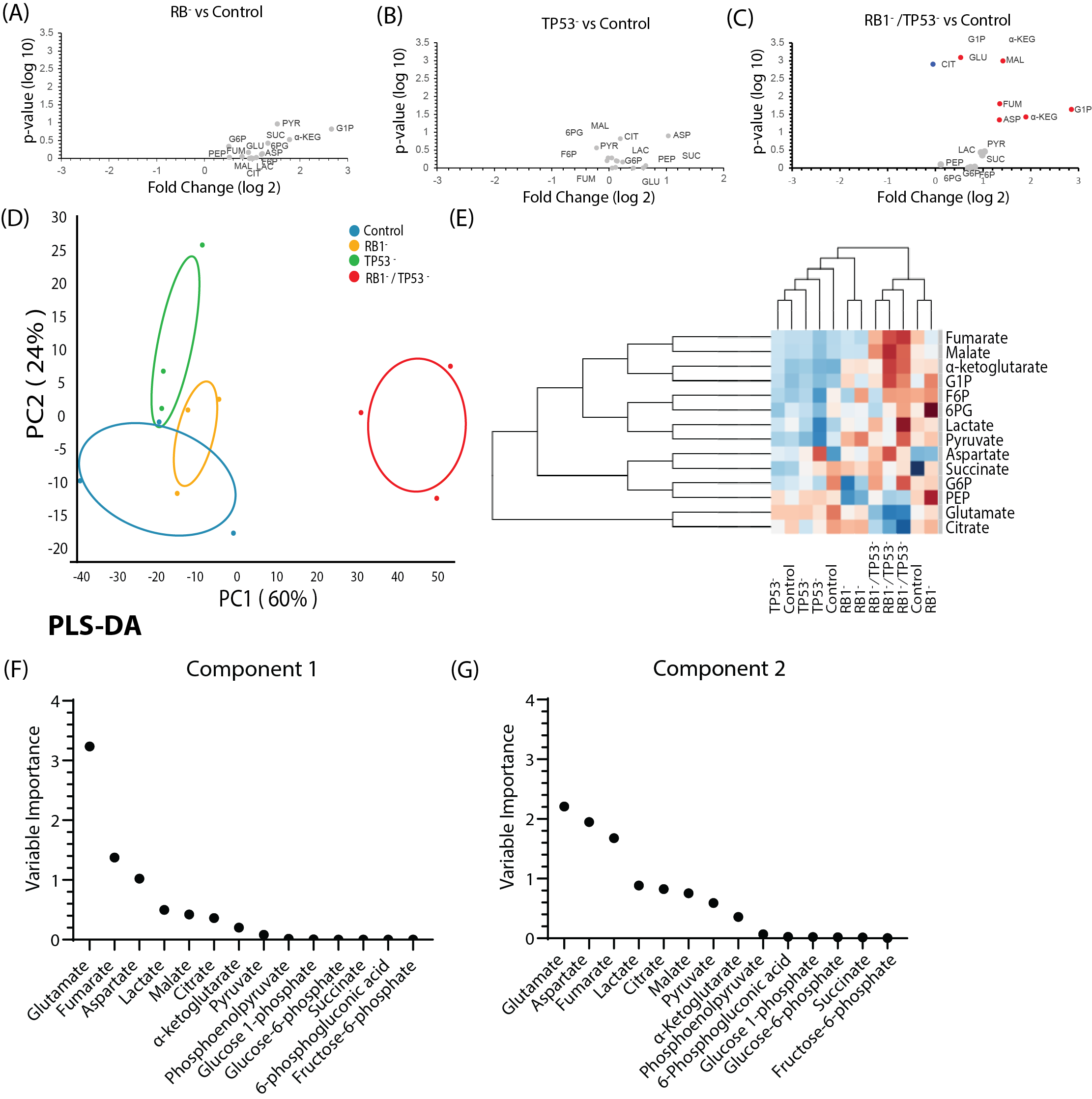


**Supplementary Figure 8****. Metabolite concentrations from IC-MS of LnCaP monoclonal Crispr KO cells relative to control.**  **(A to C)** Volcano plot of the log 2-fold change versus the associated p-value. Blue is significantly upregulated; red is significantly upregulated with **(D)** Principal component plot of the normalized concentrations. The primary separation is the RB1/TP53- dual knockout from the others. **(E)** Heat map with hierarchal clustering **(F and G)** Variable importance for the first two components in a PLS-DA classification model for the four samples. The metabolites in the TCA cycle primarily separate the samples.


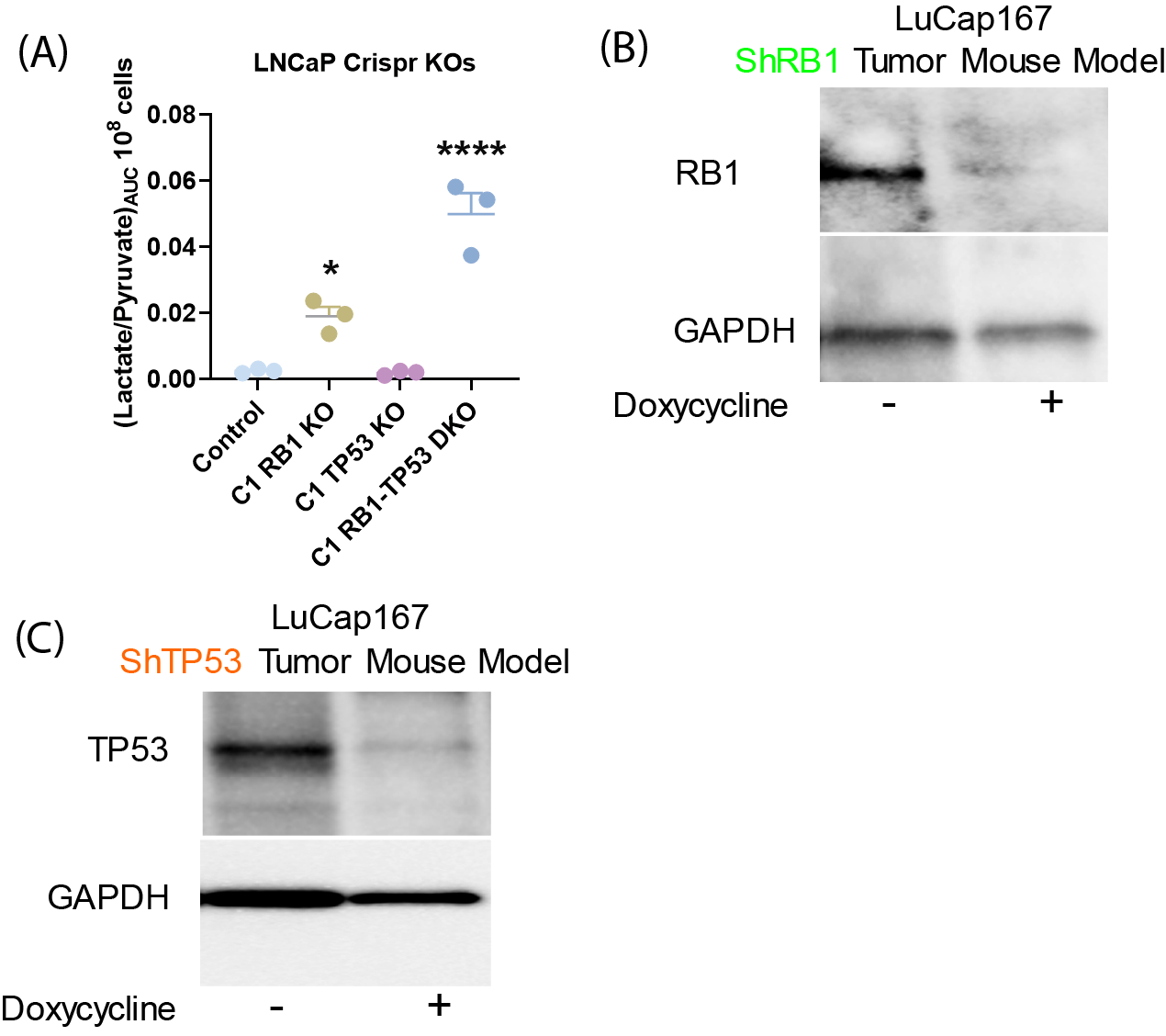


**Supplementary Figure 9****. *RB1* alone or combined with *TP53* knockdown increases LDH flux detected *in vitro* by ^13^C-NMRS in LnCaP . (A)** Dot plot of the lactate/pyruvate conversion ratio for genetically modified *RB1* and/or *TP53* LNCaP Crispr knockout models in vitro. **(B and C)** Western blot of RB1 (B) and TP53 (C) protein levels in LuCaP167 organoid-derived PDX tumors with or without *in vivo* doxycycline induction of ShRNA as described in methods.
